# Immuntherapie gegen Gliome

**DOI:** 10.1007/s00115-023-01590-5

**Published:** 2024-01-02

**Authors:** Katharina Sahm, Tobias Weiss

**Affiliations:** 1 Neurologische Klinik, Medizinische Fakultät Mannheim, MCTN, Universität Heidelberg, Mannheim, Deutschland; 2 Klinische Kooperationseinheit Neuroimmunologie und Hirntumorimmunologie, Deutsches Krebsforschungszentrum Heidelberg, Heidelberg, Deutschland; 3 Klinik für Neurologie und klinisches Neurozentrum, Universitätsspital Zürich und, Universität Zürich, Frauenklinikstr. 26, 8091 Zürich, Schweiz

**Keywords:** Immun-Checkpoint-Inhibition, Virustherapie, Hirntumorvakzine, Zelltherapie, Zytokine, Immune checkpoint inhibitors, Viral treatment, Brain tumor vaccines, Cell therapy, Cytokines

## Abstract

**Hintergrund:**

Gliome sind die häufigsten bösartigen primären Hirntumoren bei Erwachsenen. Trotz multimodaler Therapiekonzepte aus Chirurgie, Bestrahlung und Chemotherapie bleibt ihre Prognose schlecht und sie sind nicht heilbar. Neue Erkenntnisse über die Interaktionen zwischen Immunsystem und zentralem Nervensystem sowie bahnbrechende Ergebnisse bei anderen Krebserkrankungen haben dazu geführt, dass verschiedene immuntherapeutische Ansätze auch gegen Gliome untersucht und teilweise spezifisch entwickelt werden.

**Ziel der Arbeit:**

Dieser Artikel bietet einen Überblick über den aktuellen Stand verschiedener immuntherapeutischer Konzepte gegen Gliome, einschließlich Vor- und Nachteilen sowie Herausforderungen. Zudem gibt er eine Übersicht über aktuell laufende Immuntherapiestudien in Deutschland und den Nachbarländern.

**Ergebnisse:**

Bisherige randomisierte Studien zu anti-PD1-Immun-Checkpoint-Inhibition, Virustherapie sowie zur Peptidvakzinierung gegen die Variante III des epidermalen Wachstumsfaktors (EGFRvIII) beim Glioblastom waren negativ bez. eines Überlebensvorteils. Andere immuntherapeutische Ansätze wie multiepitop- oder treibermutationsbasierte Vakzinierungen, zytokinbasierte Therapien und Zelltherapien haben eine gute wissenschaftliche Grundlage und zumindest frühe Studien zur Sicherheit und pharmakodynamischen Wirkung am Tumor sind vielversprechend.

**Diskussion:**

Immuntherapien gegen Gliome sollten derzeit nur im Rahmen von Studien angewendet werden. Es bestehen noch viele Wissenslücken hinsichtlich der Wirk- und Resistenzmechanismen verschiedener Immuntherapien. Begleitende translationale Forschung ist entscheidend, um diese Lücken zu schließen und effektivere Therapien zu entwickeln.

## Abstract (en)

**Background:**

Gliomas represent the most frequent malignant primary brain tumors in adults. Despite multimodal treatment concepts involving surgery, irradiation and chemotherapy, the prognosis remains poor and they are incurable. Recent insights into the interactions between the immune system and the central nervous system as well as breakthroughs in the results of other cancer types have led to the fact that various immunotherapeutic approaches against gliomas have also been investigated and in some cases specifically developed.

**Objective:**

This article provides an overview of the current status of different immunotherapeutic concepts against gliomas, highlighting the advantages, disadvantages, and challenges. Additionally, it provides an overview of currently ongoing immunotherapeutic clinical trials in Germany and neighboring countries.

**Results:**

Previous randomized studies on antibodies against programmed cell death protein 1 (anti-PD1) immune checkpoint inhibition, viral treatment and peptide vaccination targeting the variant III of the epidermal growth factor receptor (EGFRvIII) in glioblastomas were negative with respect to survival benefits. Conversely, other immunotherapeutic approaches, such as multivalent or driver mutation-based vaccinations, cytokine-based therapy and cell therapy, demonstrated a robust scientific foundation, with at least early studies showing promising safety and pharmacodynamic effects on the tumors.

**Discussion:**

Currently, immunotherapies against gliomas should only be applied within the framework of well-designed clinical studies. There are still many knowledge gaps regarding the mechanisms of action and resistance of various immunotherapies. Accompanying translational research is essential to address these gaps and develop more effective therapies.

## Hintergrund

**Figure 1:**
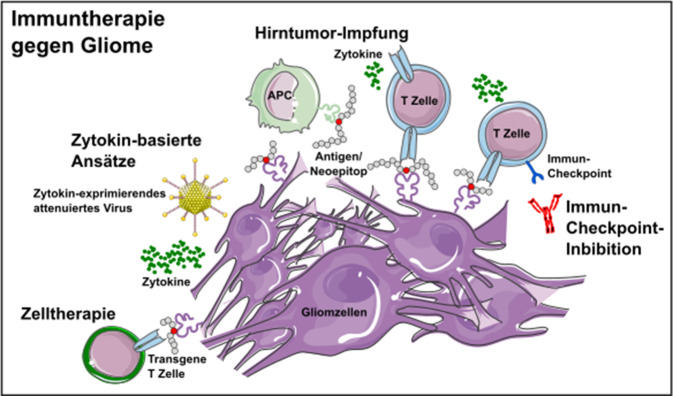

Gliome zählen zu den häufigsten primären Hirntumoren. Trotz Therapie mittels Chirurgie, Radiotherapie und Chemotherapie sind Rezidive die Regel und die Prognose bleibt schlecht. Krebsimmuntherapien haben das Ziel, angeborene und/oder erworbene Immuneffektormechanismen gegen Krebszellen zu richten. Sie haben bereits bei extrakraniellen Tumorerkrankungen zu bahnbrechenden Erfolgen geführt, weshalb verschiedene immuntherapeutische Strategien auch für Gliome untersucht werden. Grundsätzlich unterscheidet man Ansätze, die entweder direkt lokal im Tumor oder zunächst peripher und dann sekundär im Tumor immunstimulierend wirken. Des Weiteren unterscheidet man antigenspezifische von unspezifisch immunstimulierenden Ansätzen. Konkrete aktuelle Konzepte umfassen Immun-Checkpoint-Inhibition, Virustherapie, Vakzinierungen, zytokinbasierte Strategien und Zelltherapien (Abb. 1). Im Folgenden werden diese Therapiekonzepte genauer erläutert und die aktuelle Datenlage übersichtlich dargestellt.

## Immun-Checkpoint-Inhibition

Immun-Checkpoints sind Moleküle auf der Oberfläche von T‑Lymphozyten, die deren Effektorfunktion regulieren. In der Krebstherapie werden Antikörper gegen diese Oberflächenproteine eingesetzt, um die Immunantwort gegen den Tumor zu verstärken. Zu den häufigsten auch in der Neuroonkologie eingesetzten Immun-Checkpoint-Inhibitoren (ICI) gehören Antikörper gegen „programmed cell death protein 1“ (PD1) bzw. den zugehörigen Liganden PD-L1 sowie gegen „cytotoxic T‑lymphocyte-associated protein 4“ (CTLA-4).

Während der Einsatz von ICI bei systemischen Tumoren teilweise zu beeindruckenden Therapieerfolgen und einer Zulassung bereits in der Erstlinienbehandlung beispielsweise des metastasierten Melanoms geführt hat, blieben Phase-3-Studien zum Einsatz von Anti-PD1-Antikörpern zur Behandlung des Glioblastoms trotz zufriedenstellender Verträglichkeit sowohl in der Erstlinienbehandlung als auch in der Rezidivtherapie negativ [1–3]. Möglicherweise ist die unterschiedliche Mutationslast und daraus resultierende Immunogenität verschiedener Tumorentitäten eine Erklärung für das differenzielle Therapieansprechen [4], wobei eine hohe Mutationslast alleine nicht ausreicht, um ein Therapieansprechen vorherzusagen [5]. Übereinstimmend damit zeigte eine entitätsübergreifende Studie zur Behandlung Mismatch-repair-defizienter solider Tumoren mit dem Anti-PD1-Antikörper Pembrolizumab auch bei progredienten Krebserkrankungen einen therapeutischen Benefit, wobei die Effekte bei Gliomen im Vergleich zu systemischen Tumoren geringer zu sein scheinen [6]. Aufgrund dieser Studienergebnisse erhielt Pembrolizumab im Juni 2020 in den USA die Zulassung zur Behandlung rezidivierender hypermutierter Glioblastome. In Deutschland sind für einen Einsatz von ICI außerhalb klinischer Studien eine interdisziplinäre Indikationsstellung durch das neuroonkologische Tumorboard und individuelle Kostenübernahmegesuche an die zuständige Krankenkasse notwendig.

ICI könnte mit anderen T‑ und NK-Zell-basierten Therapieansätzen kombiniert werden

Eine weitere Strategie für den Einsatz von ICI bei malignen Gliomen kann auch die Definition des Zeitpunkts sein, z. B. neoadjuvant vor geplanter Reresektion [7]. Zudem ist es denkbar, ICI mit anderen T‑ und NK-Zell-basierten Therapieansätzen im Rahmen klinischer Studien zu verwenden. Aktuell werden beispielsweise in mehreren Phase-1-Studien Impfstoffe eingesetzt, um eine gegen den Tumor gerichtete T‑Zell-Antwort auszulösen, die durch den zusätzlichen Einsatz eines ICI verstärkt werden soll (NCT03893903, NCT04808245). Obwohl bisher keine Sicherheitsbedenken für Kombinationsbehandlungen festgestellt wurden, fehlen noch Ergebnisse zur Wirksamkeit. Darüber hinaus werden klinische Studien zur Identifikation prädiktiver Marker für ICI beitragen. Es werden auch neuere ICI-Strategien, die bereits erfolgreich bei systemischen Tumoren eingesetzt wurden, für Gliome geprüft, wie beispielsweise solche, die gegen das Lymphozytenaktivierungsgen 3 (LAG3) oder den T‑Zell-Immunrezeptor mit Immunglobulin- und ITIM-Domänen (TIGIT) gerichtet sind.

## Virustherapie

Bei dieser Therapieform werden onkolytische oder nichtonkolytische gentechnisch veränderte Viren eingesetzt. Diese Viren sollen selektiv oder bevorzugt Tumorzellen befallen, diese abtöten und gleichzeitig eine Immunantwort auslösen, indem sie Antigene freisetzen und Erkennungsrezeptoren des angeborenen Immunsystems aktivieren. Trotz einzelner vielversprechender Resultate in Phase-1-Studien [8] konnte in einer randomisierten Phase-3-Studie bisher kein Überlebensvorteil nachgewiesen werden [9].

## Hirntumorvakzine

Hirntumorimpfstoffe haben zum Ziel, eine gegen den Tumor gerichtete spezifische T‑Zell-Antwort auszulösen und aufrechtzuerhalten. Es gibt verschiedene Impfstofftypen, wie Peptidimpfstoffe und RNA-/DNA-Impfstoffe, die auf charakteristische Tumorantigene abzielen, sowie autologe dendritische Zellen (DC), die außerhalb des Körpers mit Peptiden oder Tumorlysat beladen werden können. DC-Impfstoffe sind aufwendiger in der Herstellung, können aber potenziell sehr viele Antigene eines Tumorlysats präsentieren. Im Gegensatz dazu zielen Peptidimpfstoffe in der Regel auf einzelne rekurrente Neoepitope ab oder werden im Fall von Multipeptidimpfstoffen nach dem Baukastenprinzip basierend auf der Antigen-Landschaft jedes Tumors und den „Major-histocompatibility-complex“(MHC)-Charakteristika jedes einzelnen Patienten individuell zusammengestellt.

Ein Beispiel für einen solchen Multipeptidimpfstoff, der auf dem MHC-Klasse-I-präsentierten Immunpeptidom basiert, ist IMA950. Die Ergebnisse einer First-in-human-Studie zur Anwendung bei Patienten mit neu diagnostiziertem Glioblastom zeigten ermutigende Ergebnisse hinsichtlich Sicherheit und Immunogenität von IMA950 in Kombination mit der Standardradiochemotherapie [10], Ergebnisse zur Effektivität stehen jedoch aus. Ein weiteres Beispiel ist ein patientenindividualisierter Multipeptidansatz, der zumindest machbar, jedoch insgesamt sehr aufwendig ist [11]. Ebenfalls auf eine Kombination aus Tumorantigenen zielt der Impfstoff ICT-107 ab, wobei hier autologe DCs mit den Impfpeptiden beladen werden. Eine randomisierte, placebokontrollierte Phase-2-Studie zeigte eine Assoziation zwischen dem Vorhandensein einer impfinduzierten Immunantwort und dem Überleben, wobei sich der Überlebensvorteil insbesondere bei HLA-A2-positiven Patienten zeigte [12], einer Kohorte, auf die MHC-Klasse-I-Impfstoffe aufgrund der Antigenrestriktion, welche die Limitierung der Antigenpräsentation auf ganz bestimmte und patientenspezifische MHC-Moleküle beschreibt, typischerweise beschränkt sind.

Impfstoffe gegen Neoepitope aus rekurrenten Treibermutationen könnten einen Vorteil bieten

Im Gegensatz zu diesen individuellen Impfstoffen, zielte der Peptidimpfstoff Rindopepimut® auf ein rekurrentes Glioblastomneoepitop ab, der Variante III des epidermalen Wachstumsfaktors (EGFRvIII). Während Phase-2-Studien zwar ein akzeptables Sicherheitsprofil und die Induktion einer antigenspezifischen Immunantwort zeigten, konnte die Effektivität bezogen auf das Überleben in einer randomisierten Phase-3-Studie nicht bestätigt werden [13]. Als mögliche Erklärung wurde die subklonale Expression von EGFRvIII diskutiert sowie der spontane Verlust dieses Antigens im Krankheitsverlauf. Impfstoffe gegen Neoepitope aus rekurrenten Treibermutationen könnten hier einen Vorteil bieten. Beispiele hierfür sind Peptidimpfstoffe gegen die Isozitratdehydrogenase-1(IDH1)-R132H-Mutation, welche IDH-mutierte diffuse Gliome charakterisiert [14], sowie die Histon-3(H3)-K27M-Mutation, die für hochaggressive diffuse Mittelliniengliome diagnoseweisend ist [15].

Auf Basis einer vielversprechenden Phase-1-Studie zur Anwendung des IDH1-Impfstoffes bei Patienten mit neudiagnostizierten IDH-mutierten Gliomen [16] untersucht aktuell eine Folgestudien an den acht Zentren des deutschen Konsortiums für translationale Krebsforschung die Sicherheit und Immunogenität des Impfstoffes in Kombination mit ICI bei rezidivierten, resezierbaren IDH-mutierten Gliomen (NCT03893903). Ein ähnliches Konzept verfolgt die INTERCEPT-H3-Studie (NCT04808245), welche neben translationalen Parametern ebenfalls Sicherheit und Immunogenität eines H3K27M-Impfstoffes in Kombination mit der Standardradiotherapie sowie dem Anti-PD-L1-Antikörper Atezolizumab untersucht. Einen anderen Ansatz verfolgt die Therapie DCVax, die nicht gegen vordefinierte Antigene gerichtet ist, sondern aus mit Tumorzelllysat beladenen autologen DCs besteht. Die kürzlich publizierte Phase-3-DCVax-L-Studie war formal positiv hinsichtlich des Überlebens, jedoch ist das Studiendesign sehr umstritten [17].

## Zytokinbasierte Ansätze

Zytokine sind lösliche oder membranständige Botenstoffe, die von verschiedenen Zellen gebildet werden und para-, auto- oder auch endokrin wirken können. Sie modulieren zahlreiche biologische Prozesse, wie Zellproliferation, Zelldifferenzierung, Zelltod und Mechanismen des angeborenen oder erworbenen Immunsystems. In der Krebsimmuntherapie werden vor allem proinflammatorische Zytokine wie Interleukine (IL‑2, IL-12, IL-15), Tumornekrosefaktor (TNF) oder Granulozyten-Monozyten-kolonienstimulierender Faktor (GM-CSF) eingesetzt, sowohl als Monotherapie als auch in Kombination mit anderen Behandlungen. Die Herausforderungen zytokinbasierter Therapien liegen in ihrer kurzen Halbwertszeit im Blut, den dosisabhängigen tumorhemmenden oder sogar tumorfördernden Eigenschaften und dem engen therapeutischen Fenster – einem begrenzten Dosierungsbereich, in dem sie wirksam, aber nicht toxisch sind. Denn insbesondere bei systemischer Zirkulation können Zytokine Begleitreaktionen hervorrufen, wie Fieber, Schüttelfrost und Blutdruckabfall bis hin zum Schock. Um diese zu verhindern, werden verschiedene Verabreichungs- und Modifikationsstrategien von Zytokinen genutzt. Im Kontext von Gliomen unterscheidet man lokale intratumorale und systemisch-intravenöse Therapiestrategien.

Zwei US-amerikanische Phase-1-Studien haben bereits die Sicherheit einer lokalen intratumoralen vektorbasierten Zytokintherapie bei Patienten mit rezidivierendem Glioblastom gezeigt [18, 19]. Dabei wurde ein attenuiertes, genetisch modifiziertes Adenovirus während der Operation stereotaktisch intratumoral in die Resektionshöhle verabreicht, um ein Genexpressionsystem in die Zellen einzuschleusen, dass nach oraler Einnahme der synthetischen Substanz Veledimex zur lokalen Produktion von IL-12 führte. Neben der akzeptablen Verträglichkeit als Monotherapie oder in Kombination mit Anti-PD1-Immun-Checkpoint-Inhibition zeigten begleitende translationale Untersuchungen, dass diese Behandlung zu einer Zunahme an T‑Zellen und anderer proinflammatorischer Signalwege im Tumor führte. Ein Nachteil dieser Strategie besteht darin, dass sie nur für Patienten infrage kommt, deren Tumor erneut operiert werden kann.

Ein vielversprechendes Immunzytokin bei Gliomen ist L19TNF

Ein anderer in Europa getesteter Ansatz beruht auf der systemischen intravenösen Gabe sog. Immunzytokine. Diese sind Antikörper-Zytokin-Fusionsproteine, die nach intravenöser Applikation zu einer lokalen Anreicherung proinflammatorischer Zytokine in Tumoren führen. Ein vielversprechendes Immunzytokin bei Gliomen ist L19TNF, das aus dem Antikörper L19 besteht, der spezifisch ein Antigen im Stroma und den Blutgefäßen von Tumoren bindet, und TNF. In präklinischen Gliom-Mausmodellen zeigte es vielversprechende Antitumoraktivität sowohl als Monotherapie als auch in Kombination mit Radiochemotherapie oder Lomustin [20, 21]. Erste präliminäre Daten bei Patienten zeigen zudem, dass es sicher ist, eine akzeptable Verträglichkeit und vielversprechende Aktivität aufweist. Eine Phase-1/2-Studie, die L19TNF als Monotherapie bei Patienten mit erstem Glioblastomrezidiv nach Standardtherapie untersuchte, hat kürzlich die Rekrutierung abgeschlossen, und die Ergebnisse werden in Kürze erwartet. Aktuell laufen zwei weitere Phase-1/2-Studien, bei denen L19TNF entweder in Kombination mit Radiochemotherapie bei Patienten mit neu diagnostiziertem Glioblastom oder in Kombination mit Lomustin bei Patienten mit Glioblastomrezidiv untersucht wird. Letztere Studie hat kürzlich die multizentrische Phase in verschiedenen Zentren in Deutschland, der Schweiz, Frankreich und Italien gestartet.

## Zelltherapien

Bei den meisten aktuellen Zelltherapien werden Immunzellen aus dem peripheren Blut isoliert, im Labor mittels viraler, nichtviraler Vektoren oder mRNA-Technologie so verändert, dass sie Tumorantigene erkennen, und anschließend wieder in den Körper zurückgeführt. Die Veränderungen umfassen vor allem synthetische Rezeptoren, bekannt als chimäre Antigenrezeptoren (CAR), die aus einer tumorbindenden Domäne und einer oder mehreren immunzellaktivierenden Domänen bestehen, sowie natürlich vorkommende T‑Zell-Rezeptoren. Die meisten Zelltherapien im Gliomkontext richten sich gegen einzelne Tumorantigene, wie z. B. IL13Rα2, HER2, EGFRvIII, GD2 [22–25]. Dies birgt die Gefahr, dass die Tumorzellen das Antigen herunterregulieren, um der Immunabwehr zu entgehen.

Die „tumor inflammation-associated neurotoxicity“ ist spezifisch für die Behandlung von Hirntumoren

Die Studienlage für Zelltherapien im Kontext von Gliomen ist limitiert, aber am weitesten fortgeschritten zu CAR-T-Zellen beim Glioblastom. Eine verfügbare Phase-1-Studie hat die Sicherheit von CAR-T Zellen bei Patienten mit H3K27M-mutierten diffusen intrinsischen Pons- oder spinalen Gliomen untersucht [26]. Die CAR-T-Zell-Therapie scheint sowohl intravenös als auch intratumoral sicher zu sein, und das Auftreten schwerer behandlungsassoziierter Toxizitäten, wie das Zytokinfreisetzungssyndrom oder das immuneffektorzellassoziierte Neurotoxizitätssyndrom, ist im Vergleich zu hämatologischen Erkrankungen deutlich seltener [27]. Eine kürzlich beschrieben besondere Nebenwirkung, die spezifisch für die Behandlung von Hirntumoren ist, ist die „tumor inflammation-associated neurotoxicity“ (TIAN), die auf einer Zunahme der lokalen tumorbedingten Raumforderung oder einer lokalen neuronalen Dysfunktion beruht und zu fokalen Symptomen bis hin zu erhöhtem Hirndruck führen kann [28]. Bei dieser Nebenwirkung sollten neben Immunsuppressiva auch mechanische Therapien wie die Liquordrainage evaluiert werden. Insgesamt ist die Aktivität von CAR-T-Zellen bei Gliomen begrenzt, und es sind dringend randomisierte Studien erforderlich, um den Nutzen hinsichtlich des Überlebens zu untersuchen.

Als Alternative zu modifizierten T‑Zellen hat eine kürzlich durchgeführte multizentrische Phase-1-Studie die Sicherheit intratumoral injizierter CAR-NK-Zellen gezeigt [29]. Allerdings wurde hier eine Zelllinie verwendet, die vor der Injektion bestrahlt werden musste, was zu einer zeitlich sehr limitierten Persistenz dieser Zellen führte. Derzeit werden Patienten in diese Studie aufgenommen, um die Sicherheit der intratumoral injizierten CAR-NK-Zellen in Kombination mit dem Anti-PD1-Antikörper Ezabenlimab zu untersuchen (NCT03383978; Tab. 1).

**Table 1:** 

Titel der Studie	Phase	Intervention	Tumorentität	Identifier
*Hirntumorvakzine und Immun-Checkpoint-Inhibition*				
AMPLIFYing NEOepitope-specific VACcine Responses in Progressive Diffuse Glioma	1	IDH1R132H Peptidimpfstoff und/oder Avelumab	Rezidivierte, resektable IDH-mutierte Gliome	NCT03893903
A MultIceNTER Phase I Peptide VaCcine Trial for the Treatment of H3-Mutated Gliomas	1	H3K27M Peptidimpfstoff und Atezolizumab	Neudiagnostizierte H3K27M-mutierte Gliome	NCT04808245
Multi Peptide Vaccination With XS15 in Addition to Standard Postoperative Radiation Therapy and Temozolomide Chemotherapy in Newly Diagnosed Glioblastoma	1	Multipeptidvakzin XS15	Neudiagnostizierte Glioblastome	NCT04842513
Efficiency of Vaccination With Lysate-loaded Dendritic Cells in Patients With Newly Diagnosed Glioblastoma	2	Tumorlysatbeladene DCs	Neudiagnostizierte Glioblastome	NCT03395587
Safety and Tolerability of CVGBM in Adults With Newly Diagnosed MGMT-Unmethylated Glioblastoma or Astrocytoma	1	CV09050101 mRNA-Vakzin	Neudiagnostizierte MGMT unmethylierte Glioblastome	NCT05938387
*Zytokinbasierte Ansätze*				
Safety and Efficacy of L19TNF Plus Temozolomide Chemoradiotherapy in Patients With Newly Diagnosed Glioblastoma	1/2	Immunzytokin L19TNF und Radiochemtherapiy	Neudiagnostizierte Glioblastome	NCT04443010
A Study to Evaluate Safety and Efficacy of L19TNF Plus Lomustine in Patients With Glioblastoma at First Progression	1/2	Immunzytokin L19TNF und Lomustin	Erstes Glioblastomrezidiv	NCT04573192
*Zelltherapien*				
Intracranial Injection of NK-92/5.28.z Cells in Combination With Intravenous Ezabenlimab in Patients With Recurrent HER2-positive Glioblastoma	1	HER2-spezifische CAR-NK-Zellen und Ezabenlimab	Progrediente HER2-positive Glioblastome	NCT03383978

## Fazit für die Praxis

- Bisher konnte bei Gliomen kein Überlebensvorteil durch Immuntherapie nachgewiesen werden.
- Der Stellenwert von Immun-Checkpoint-Inhibition (ICI) bei Gliomen ist nicht definiert. Nach den negativen Phase-3-Studien in der Erstlinientherapie des Glioblastoms gibt es in aktuellen klinischen Studien das Konzept, den Zeitpunkt der ICI neu zu definieren (z. B. neoadjuvant) bzw. ICI in Kombination mit anderen Immuntherapien zu verwenden.
- Aktuell laufende Studien befassen sich mit der Sicherheit und Effektivität neoepitopbasierter Vakzine bei IDH- und H3K27M-mutierten Gliomen sowie der Anwendung von CAR-T- und CAR-NK-Zellen oder Immunzytokinen bei Patienten mit Glioblastom.
